# In situ formation of photoactive B-ring reduced chlorophyll isomer in photosynthetic protein LH2

**DOI:** 10.1038/s41598-020-76540-1

**Published:** 2020-11-09

**Authors:** Yoshitaka Saga, Yuji Otsuka, Daichi Funakoshi, Yuto Masaoka, Yu Kihara, Tsubasa Hidaka, Hiroka Hatano, Hitoshi Asakawa, Yutaka Nagasawa, Hitoshi Tamiaki

**Affiliations:** 1 Department of Chemistry, Faculty of Science and Engineering, Kindai University, Higashi-Osaka, Osaka 577-8502 Japan; 2 Graduate School of Life Sciences, Ritsumeikan University, Kusatsu, Shiga 525-8577 Japan; 3 Graduate School of Natural Science and Technology, Kanazawa University, Kanazawa, 920-1192 Japan; 4 Nanomaterials Research Institute, Kanazawa University, Kanazawa, 920-1192 Japan

**Keywords:** Biochemistry, Organic chemistry, Biophysical chemistry, Proteins

## Abstract

Natural chlorophylls have a D-ring reduced chlorin π-system; however, no naturally occurring photosynthetically active B-ring reduced chlorins have been reported. Here we report a B-ring reduced chlorin, 17,18-didehydro-bacteriochlorophyll (BChl) *a*, produced by in situ oxidation of B800 bacteriochlorophyll (BChl) *a* in a light-harvesting protein LH2 from a purple photosynthetic bacterium *Phaeospirillum molischianum*. The regioselective oxidation of the B-ring of B800 BChl *a* is rationalized by its molecular orientation in the protein matrix. The formation of 17,18-didehydro-BChl *a* produced no change in the local structures and circular arrangement of the LH2 protein. The B-ring reduced 17,18-didehydro-BChl *a* functions as an energy donor in the LH2 protein. The photoactive B-ring reduced Chl isomer in LH2 will be helpful for understanding the photofunction and evolution of photosynthetic cyclic tetrapyrrole pigments.

## Introduction

**Figure 1:**
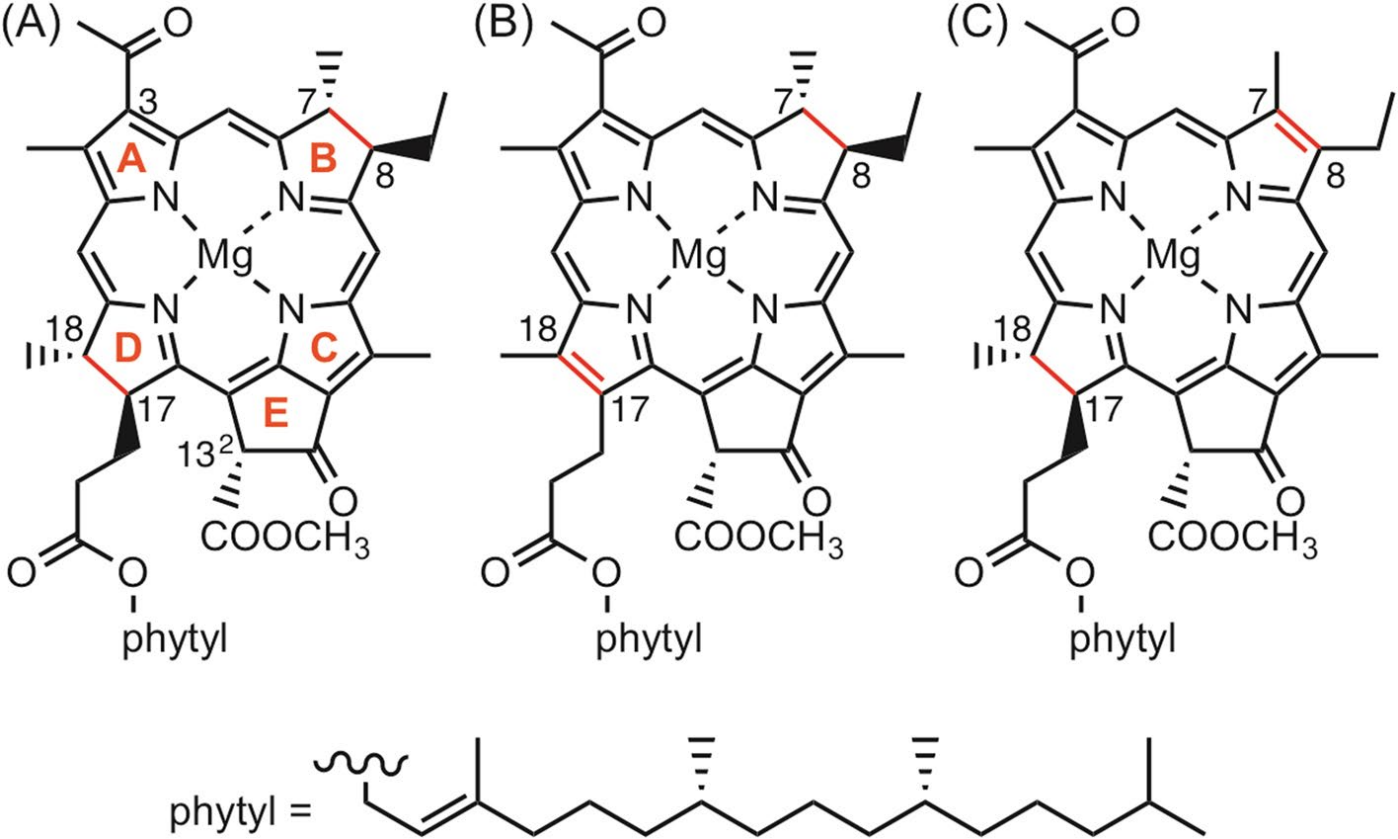
Molecular structures of BChl *a* (**A**), 17,18-didehydro-BChl *a* (**B**, denoted as **1**), and 3-acetyl-Chl (AcChl) *a* (**C**).

Cyclic tetrapyrroles with modified skeletons and peripheral groups have essential roles in various biofunctional proteins^1–3^. Chlorophyll (Chl) molecules, involved in the solar-energy conversion processes of oxygenic photosynthesis, typically contain an unsymmetrical conjugated tetrapyrrole π-system, in which the C17–C18 bond in the D-ring is hydrogenated^1,4–8^. The D-ring reduced chlorin (17,18-dihydroporphyrin) skeleton is responsible for efficient light absorption in the visible portion of the solar spectrum. The in vivo conversion from the porphyrin macrocycle in the precursor of Chls, protochlorophyllide *a*, to the D-ring reduced chlorin ring is regio- and stereoselectively mediated by protochlorophyllide oxidoreductase (POR)^5–8^. In photosynthetic bacteria, further hydrogenation of the C7 = C8 double bond in the B-ring of the chlorin macrocycle is mediated by chlorophyllide oxidoreductase (COR) to produce a bacteriochlorin (7,8,17,18-tetrahydroporphyrin), which leads to bacteriochlorophyll (BChl) *a* (Fig. 1A)^5–10^. However, it remains unclear why natural phototrophs select D-ring reduced chlorin and both B- and D-rings reduced bacteriochlorin for light-harvesting and charge separation processes.

B-ring reduced chlorin (7,8-dihydroporphyrin), in which the position of the hydrogenation in the cyclic tetrapyrrole is diagonally opposite to that in naturally occurring D-ring reduced chlorins, is a fascinating Chl isomer^11,12^. However, no photosynthetically active B-ring reduced chlorins have been discovered in nature, likely because the order of the ring reductions in the (B)Chl biosynthetic pathway is determined by the substrate specificities of POR and COR^5,13^. Substitution of D-ring reduced natural Chls with corresponding artificial B-ring reduced chlorin isomers in photosynthetic proteins provides new insights into the photofunctional mechanisms of Chl-polypeptide complexes. Additionally, this substitution might indicate the reason for selection of the D-ring reduced chlorin π-system in photosynthetic evolution. Herein, we report the in situ formation of a B-ring reduced chlorin pigment, 17,18-didehydro-BChl *a* (**1**, Fig. 1B), from BChl *a* in light-harvesting protein LH2 from a purple photosynthetic bacterium *Phaeospirillum molischianum* (denoted as *molischianum*-LH2) by treatment of this protein with 2,3-dichloro-5,6-dicyano-1,4-benzoquinone (DDQ).

LH2 proteins are light-harvesting pigment-polypeptide complexes in purple photosynthetic bacteria. The BChl *a* pigments are circularly arranged by support of transmembrane α- and β-polypeptides in LH2 proteins^14–16^. BChl *a* pigments in LH2 are classified into two types, B800 and B850, on the basis of the peak positions of their lowest energy absorption bands called Q_y_ bands. Intracomplex excitation energy transfer (EET) occurs efficiently from B800 to B850 BChl *a*^17,18^.

Here, we focus on B800 BChl *a*, which is sandwiched between the outer β-polypeptides in LH2 proteins. In *molischianum*-LH2, the D-ring of B800 BChl *a* is oriented toward the surface of the protein matrix (Fig. S1A)^14^. This molecular orientation markedly differs from that in the other structural-determined LH2 protein from *Rhodoblastus acidophilus* (denoted as *acidophilus*-LH2), where the D-ring in B800 BChl *a* is embedded in the protein matrix (Fig. S1B)^15,16^. The B800 geometry in *molischianum*-LH2 shows promise for producing a photoactive B-ring reduced chlorin by selective in situ oxidation of the D-ring in B800 BChl *a*.

## Results and discussion

**Figure 2:**
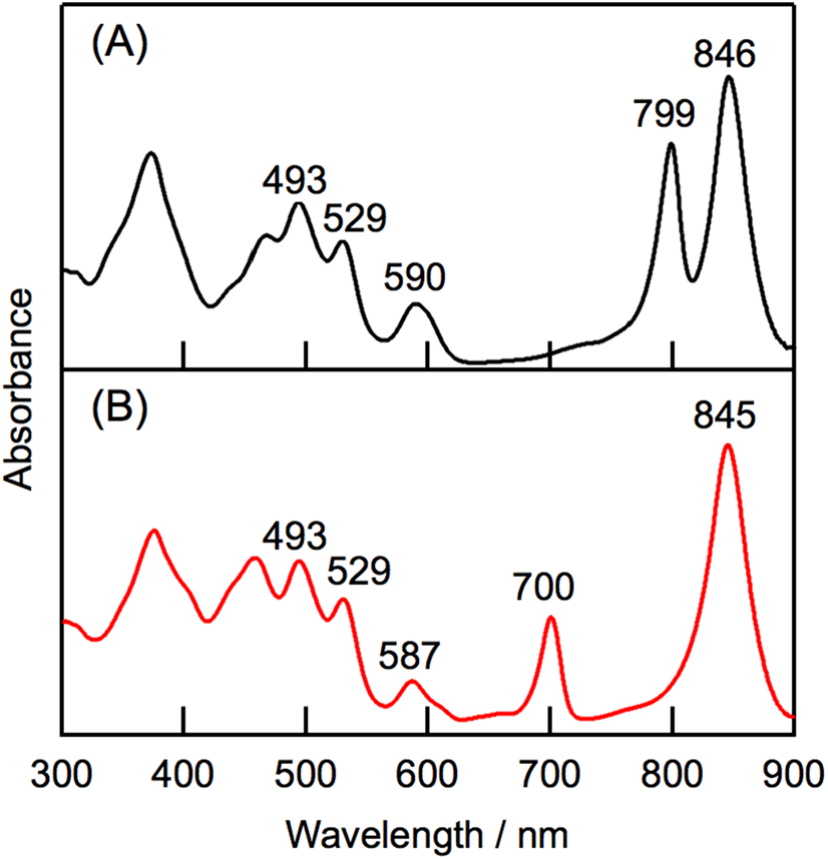
Electronic absorption spectra of native *molischianum*-LH2 (**A**) and oxidized *molischianum*-LH2 (**B**) in 20 mM Tris buffer containing 0.02% *n-*dodecyl-*β*-**D**-maltoside (pH 8.0).

Figure 2 compares the electronic absorption spectrum of *molischianum*-LH2 after incubation with DDQ (denoted as oxidized *molischianum*-LH2) with that of native LH2. A Q_y_ absorption band at 799 nm of B800 BChl *a* was absent from the oxidized *molischianum*-LH2, and a new absorption band appeared at 700 nm. We attribute this feature to the Q_y_ band of the oxidized pigment, produced from B800 BChl *a*. Difference spectrum of the oxidized/native *molischianum*-LH2 revealed a Soret band for the oxidized pigment at 449 nm (Fig. S2). In the oxidation of B800 BChl *a*, the peak position and the bandwidth of the Q_y_ band of B850 BChl *a* remained unchanged. The absorption spectrum of oxidized *molischianum*-LH2 barely changed by incubation with sodium ascorbate (100 mM), indicating the oxidation of B800 BChl *a* is irreversible (Fig. S3).

Spectral changes of oxidized *molischianum*-LH2 at 40 °C are compared with those of native LH2 to check the stability of oxidized LH2. Both the LH2 samples exhibited no change in their spectra (Fig. S4). These results suggest that oxidized *molischianum*-LH2 is stable in a similar level to native LH2. The stability of oxidized *molischianum*-LH2 is also confirmed by no degradation of oxidized *molischianum*-LH2 during its purification and various measurements.

**Figure 3:**
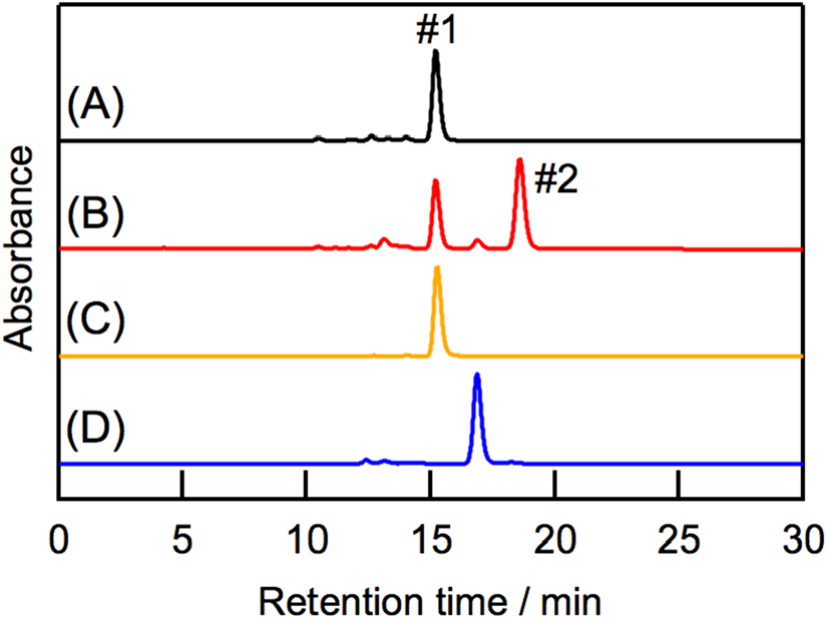
HPLC chromatograms of chlorophyllous pigments in oxidized *molischianum*-LH2 (**A**,**B**), BChl *a* (**C**), and AcChl *a* (**D**). Chromatograms A, B, C, and D were recorded at 770, 690, 770, and 680 nm, respectively.

The Q_y_ peak position of the new pigment in oxidized *molischianum*-LH2 shifted to a longer wavelength than that of D-ring reduced 3-acetyl-Chl (AcChl) *a* (Fig. 1C) in the B800 site of *molischianum*-LH2 at 690 nm^19^. This result suggests that the newly formed pigment in the oxidized *molischianum*-LH2 was not AcChl *a*. This assumption was confirmed by high-performance liquid chromatography (HPLC) analysis and electronic absorption spectroscopy of the pigments from oxidized *molischianum*-LH2. Oxidized *molischianum*-LH2 had two major chlorophyllous pigments (Fig. 3A,B). The former pigment (#1 in Fig. 3A) was BChl *a*, which we attribute to residual B850 BChl *a* (Fig. 3C). We assigned the latter pigment (#2 in Fig. 3B) to the oxidized pigment. This oxidized pigment eluted more slowly in the HPLC analysis than AcChl *a* (Fig. 3D). In addition, the Q_y_ peak position of the oxidized pigment at 692 nm in methanol was red-shifted compared with that of AcChl *a* at 685 nm (Fig. S5). The oxidized pigment had a signal at *m*/*z* 909.5378 in high-resolution mass-spectrometry (HRMS) measurements. This value corresponds to that of the calculated value (MH^+^, 909.5375) of a didehydrogenated pigment derived from BChl *a*. These results suggest that the pigment formed through the DDQ oxidation of *molischianum*-LH2 is an isomer of AcChl *a*.

**Figure 4:**
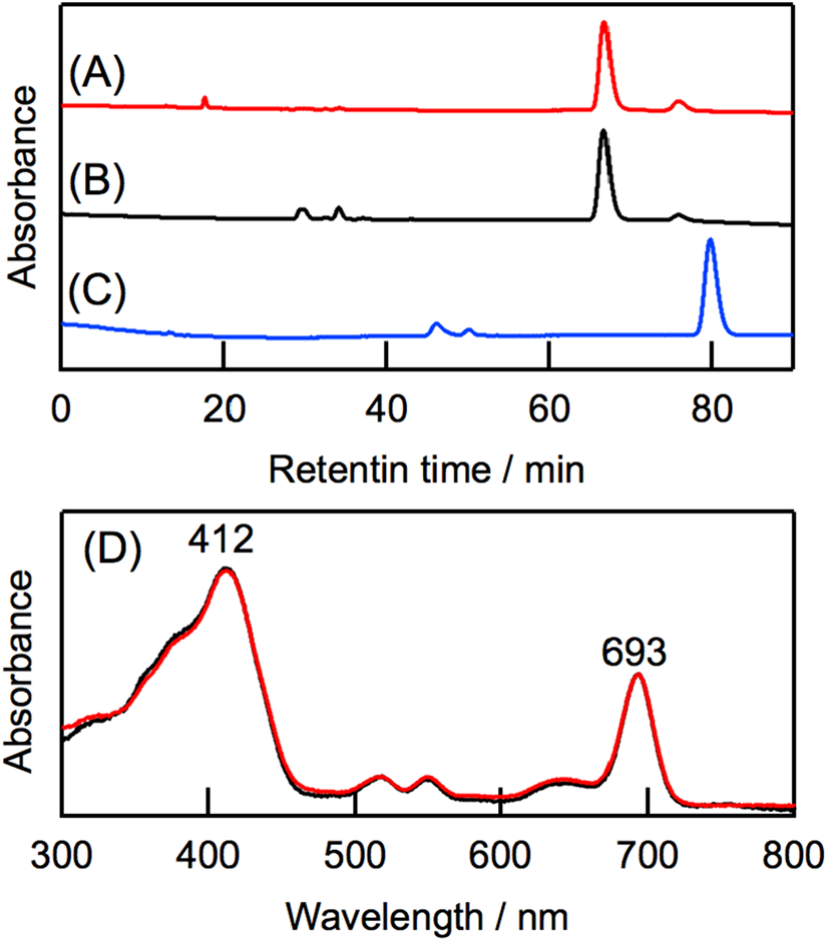
HPLC chromatograms of demetallated form of **1** derived from oxidized *molischianum*-LH2 (**A**), 17,18-didehydro-BPhe *a* (**B**), and 3-acetyl-pheophytin *a* (**C**). Chromatograms A/B and C were recorded at 690 and 680 nm, respectively. Overlapped electronic absorption spectra of the demetallated form of **1** (red) and authentic 17,18-didehydro-BPhe *a* (black) in methanol are shown in (**D**).

To assign the oxidized pigment formed in *molischianum*-LH2, the pigment was demetallated and compared with a structurally determined 17,18-didehydro-bacteriopheophytin (BPhe) *a* that was synthesized from BPhe *a* by oxidation with FeCl_3_^11,12^ (see “Materials and methods” as well as Fig. S6). Note that the removal of Mg from BChl *a* is necessary for synthesis of the B-ring reduced chlorin possessing the 13^2^-methoxycarbonyl group. HPLC analysis revealed that the retention time of the free-base derived from the oxidized pigment in *molischianum*-LH2 was identical to that of authentic 17,18-didehydro-BPhe *a* (Fig. 4A,B), but differed from that of the D-ring reduced 3-acetyl-pheophytin *a* (Fig. 4C). The electronic absorption spectrum of the oxidized pigment in methanol was identical to that of 17,18-didehydro-BPhe *a* (Fig. 4D). These results indicate that the newly formed pigment in *molischianum*-LH2 by the DDQ oxidation is 17,18-didehydro-BChl *a* (hereafter denoted as **1**).

No porphyrin-type pigment was detected in the pigments that were extracted from oxidized *molischianum*-LH2, indicating that the treatment of *molischianum*-LH2 with DDQ did not oxidize the B-ring of B800 BChl *a*. No conversion from B800 BChl *a* to the corresponding porphyrin-type pigment, 3-acetyl-protochlorophyll *a*, has been reported in the DDQ oxidation of *acidophilus*-LH2^20^. The regioselective oxidation of B800 BChl *a* observed here suggests that the polypeptides sterically protect the pyrrole ring of BChl *a* inside the protein matrix.

We confirmed the effects of the DDQ oxidation on lycopene, a major carotenoid in *molischianum*-LH2, by HPLC analysis. Lycopene from oxidized *molischianum*-LH2 was detected at the same retention time as that of native LH2 and a standard sample (Fig. S7), indicating that the lycopene in *molischianum*-LH2 was unaffected by the DDQ oxidation. The stable peak positions of lycopene in the absorption spectrum of oxidized *molischianum*-LH2 (Figs. 2 and S2) confirmed that DDQ oxidation had no effect on lycopene.

The effects of the B800 oxidation on the protein structure of *molischianum*-LH2 were examined by a frequency modulation atomic force microscopy (FM-AFM), size-exclusion chromatography (SEC), and circular dichroism (CD) spectroscopy. The crystallographic structure of LH3 protein^21^ indicates that the circular arrangement is conserved even if local interactions of BChl *a* with polypeptides; namely substitution of amino acid residues that participate in the formation of the BChl-binding pocket is proved to produce no effect on the overall structures of light-harvesting proteins from purple bacteria. In contrast, information on the effect of chemical modification of BChl *a* bound to LH2 and related proteins on their protein structures is less available. The structural analysis demonstrated here will provide useful information in this regard.

**Figure 5:**
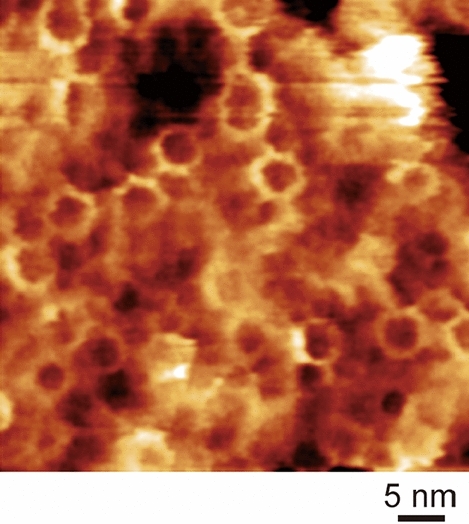
FM-AFM images of oxidized *molischianum*-LH2 on mica in 20 mM Tris buffer containing 150 mM NaCl (pH 8.0).

The ring structure of oxidized *molischianum*-LH2 was clearly visualized by FM-AFM (Fig. 5). This circular arrangement is quite similar to that of native *molischianum*-LH2^22^. The height profiles in the AFM results (Fig. S8) indicated the averaged top-to-top distance of oxidized *molischianum*-LH2 to be 4.7 ± 0.2 nm (average and standard deviation of 16 samples). This value is almost identical to that of native *molischianum*-LH2 (4.5 ± 0.5 nm)^22^. Therefore, no deformation of the LH2 ring structure was induced by the in situ oxidation of B800 BChl *a* in *molischianum*-LH2. The elution volume of oxidized *molischianum*-LH2 in the SEC chromatogram was the same as that of native *molischianum*-LH2 (Fig. S9), indicating that the protein size was unchanged by the oxidation of B800 BChl *a*.

In the CD spectrum of oxidized *molischianum*-LH2, a negative CD signal of B850 BChl *a* was observed at around 855 nm (Fig. S10C). This signal was similar to that of native *molischianum*-LH2 (Fig. S10A)^19,22^, indicating that the orientation and electronic structures of B850 BChl *a* were not influenced by the B800 oxidation. On oxidation of B800 BChl *a*, a reverse *S*-shaped signal for B800 BChl *a* at around 800 nm disappeared and a new negative CD signal appeared at around 700 nm (Fig. S10C). This negative signal is assigned to **1** in B800 sites. Note that AcChl *a* reconstituted into the B800 site of *molischianum*-LH2 barely exhibited CD signal in the Q_y_ region^19^. This difference suggests some disarrangements of reconstituted AcChl *a* in the B800 site of *molischianum*-LH2^19^, although the interactions of AcChl *a* with the surrounding amino acid residues are essentially the same as those of **1** and native B800 BChl *a*. The negative CD signal in the spectrum of oxidized *molischianum*-LH2 at around 220 nm (Fig. S10D) closely resembles that of native LH2 (Fig. S10B), indicating that the content of α-helices was unchanged by the B800 oxidation.

Excitation of oxidized *molischianum*-LH2 at 700 nm produced an emission from B850 BChl *a* at 860 nm (Fig. S11B, black curve). This emission is in line with the B850 emission by excitation of B800 BChl *a* in native LH2 (Fig. S11A, black curve). In the excitation spectra of oxidized and native *molischianum*-LH2, the bands at around 700 and 800 nm were detected, respectively (Fig. S11, red curves). These results indicate that **1** and B800 BChl *a* function as energy donors in oxidized and native *molischianum*-LH2, respectively.

**Figure 6:**
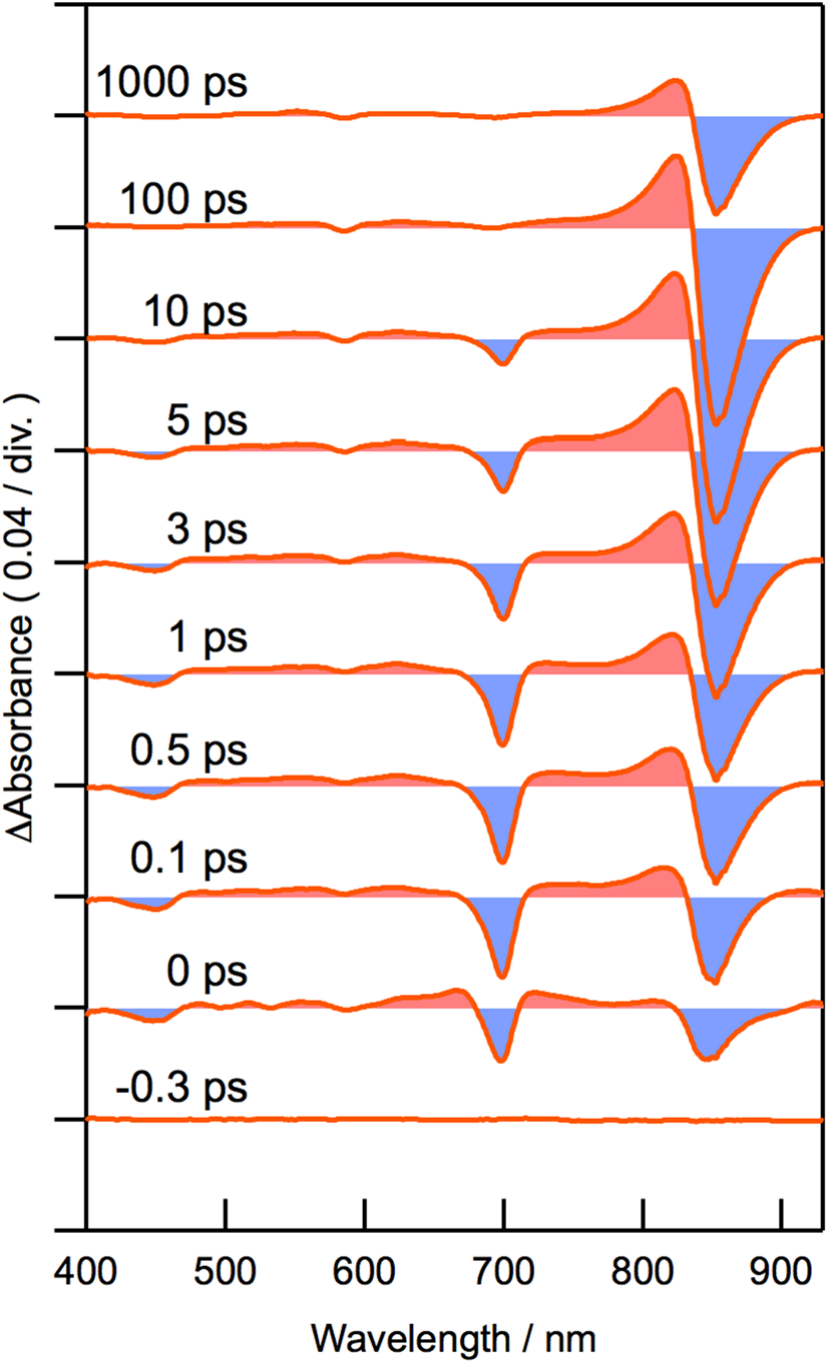
TA spectra of oxidized *molischianum*-LH2 excited at 700 nm with pulse durations of 16 fs (fwhm). The time delay increases from bottom to top.

We used femtosecond transient absorption (TA) spectroscopy to examine the EET dynamics from **1** to B850 BChl *a* in the oxidized *molischianum*-LH2 (Fig. 6). Excitation of oxidized *molischianum*-LH2 at 700 nm produced a negative band with a minimum at 700 nm, which was assigned to superposition of the ground state bleach (GSB) and stimulated emission (SE) of the energy-donating **1** in the B800 site. This negative band subsequently decayed, accompanied by new positive and negative bands at 825 and 855 nm, respectively, indicating EET from **1** to B850 BChl *a*^19,23,24^.

**Figure 7:**
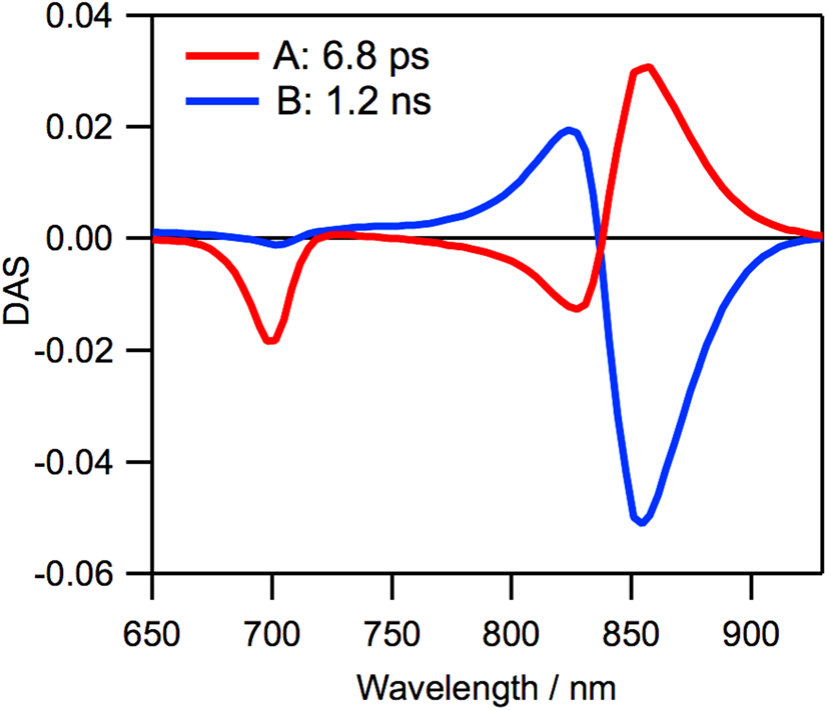
DAS obtained from the global analysis of the TA spectra of oxidized *molischianum*-LH2. Components with time constants of 6.8 ps (**A**) and 1.2 ns (**B**) were obtained.

We obtained two decay-associated spectra (DAS) (Fig. 7) by global analysis of the time dependence of the differential absorbance (ΔAbs) at various wavelengths (Fig. S12). This global analysis is in line with our previous analysis of native *molischianum*-LH2 and AcChl *a*-reconstituted *molischianum*-LH2^19^. The DAS component A with a shorter lifetime (6.8 ps) has a negative band at 700 nm, which corresponds to the decrease of the mixed GSB/SE band of **1**, with a pair of 825-nm negative and 855-nm positive bands due to the increase of the positive excited-state absorption band and the negative GSB/SE band of B850 BChl *a*, respectively. The DAS component B with a longer lifetime (1.2 ns) represents the decay of the excited state of B850 BChl *a*.

The TA spectroscopy results reveal that **1** can function as an energy donor in oxidized *molischianum*-LH2. The intracomplex EET dynamics from **1** to B850 BChl *a* is homogeneous, suggesting that the molecular orientation of **1** in the eight B800 pockets is not affected by the DDQ oxidation. The lifetime of the DAS component A, which originates from the combination of the decay of **1** with the rise of B850 BChl *a*, indicates that intracomplex EET in oxidized *molischianum*-LH2 occurs approximately sevenfold slowly relative to that in native LH2 (990 fs)^19^. This slow intracomplex EET is attributed to low spectral overlap between energy-donating **1** and energy-accepting B850 BChl *a*. The intracomplex EET in oxidized *molischianum*-LH2 was slightly slower than that in AcChl *a*-reconstituted LH2 (5.0 ps)^19^ despite a slight red-shift of the Q_y_ band of **1** in the B800 site (700 nm) compared with that of AcChl *a* (690 nm). One possible reason for this result is the difference in the electron delocalization between B-ring reduced and D-ring reduced chlorins. Density functional theory (DFT) calculations indicated that electron density was delocalized over the β-position of the D-ring in the LUMO of derivatives of **1**, although no delocalization was found at the β-positions of both the B- and D-rings in the LUMO of AcChl *a* derivatives^11^. Such a difference in the distribution of the electron densities might lead to slightly slow intracomplex EET in oxidized *molischianum*-LH2.

To summarize, a regioselective isomer of natural Chls, namely B-ring reduced chlorin, is produced by selective in situ oxidation of B800 BChl *a* in *molischianum*-LH2. The B-ring reduced chlorin functions as an antenna pigment in the LH2 protein. Regioselective dehydrogenation of the D-ring of B800 BChl *a* occurs because of its molecular orientation in *molischianum*-LH2, where the D-ring of B800 BChl *a* faces outside the protein. The position of the dehydrogenation in B800 BChl *a* in *molischianum*-LH2 by DDQ oxidation is diagonally opposite to that in *acidophilus*-LH2^20^. This different regioselectivity can be rationalized by the difference in the B800 orientations between the two types of LH2 proteins (Fig. S1), and indicates an importance of the polypeptides, which protect the pyrrole rings in BChl *a* in the protein matrix. Additionally, the regioselectivity we observe in the protein matrix contrasts with that in preferential dehydrogenation of the B-ring of BChl *a* derivatives in organic solvents^11,25^. This is the first example of a photoactive B-ring reduced chlorin pigment in photosynthetic proteins. We hope that these findings will be helpful for understanding the roles of photosynthetic cyclic tetrapyrrole pigments on the light-harvesting proteins and wrestling with an open question why nature selects D-reduced Chl pigments.

## Materials and methods

### Apparatus

Electronic absorption and CD spectra were measured with a spectrophotometer (UV-2600, Shimadzu) and a spectropolarimeter (J-820, JASCO), respectively. Fluorescence emission and excitation spectra were measured with a spectrophotometer (F-7100, Hitachi). HPLC was performed with a pump (LC-20AT, Shimadzu) and detectors (SPD-M20A and SPD-20AV, Shimadzu). SEC was performed with an ÄKTAprime plus system (GE Healthcare). ^1^H NMR spectra were measured with an NMR spectrometer (ECA-600, JEOL); chemical shifts were expressed (in ppm) relative to CHCl_3_ (7.26) as an internal reference. HRMS measurements were conducted with a spectrometer (micrOTOF II, Bruker) by atmospheric pressure chemical ionization (APCI).

### Materials

LH2 protein was isolated from the cultured cells of a purple photosynthetic bacterium *Phaeospirillum molischianum* DSM120^26^. BChl *a* was isolated from a purple photosynthetic bacterium *Rhodobacter sphaeroides*, and was converted to AcChl *a* and BPhe *a* by DDQ oxidation^27^ and demetallation under acidic conditions^28^, respectively. Lycopene and DDQ were purchased from Wako Pure Chemical Industries. A detergent *n-*dodecyl-*β*-D-maltoside (DDM) was purchased from Dojindo Laboratories.

### DDQ treatment of LH2

*Molischianum*-LH2 was treated with DDQ basically according to a previous report^20^. A solution of native *molischianum*-LH2 in 20 mM Tris buffer containing 0.1% DDM (pH 8.0) was mixed with 1/10 volume of an acetone solution of DDQ. The final concentration of DDQ was 0.2 mM. The mixed solution was incubated at 35 °C in the dark. After disappearance of the Q_y_ band of B800 BChl *a*, DDQ was removed by ultrafiltration using Amicon centricon concentrators (50 kDa cutoff, Merck Millipore). The oxidized LH2 was purified by anion-exchange column chromatography using Whatman DE52 resin (GE Healthcare). The protein was desalted by ultrafiltration using Amicon centricon concentrators (50 kDa cutoff, Merck Millipore).

### HPLC analysis of chlorophyllous pigments in LH2

Chlorophyllous pigments in *molischianum*-LH2 were analyzed by reverse-phase HPLC reported elsewhere^20^. *Molischianum*-LH2 proteins were concentrated by ultracentrifugation with Amicon centricon concentrators (50 kDa cutoff, Merck Millipore), followed by evaporation with a smart evaporator (BioChromato) in the dark. Then, chlorophyllous pigments were extracted from the samples with methanol, and were eluted on a reverse-phase column 5C_18_-AR-II (6 mm i.d. × 250 mm, Nacalai Tesque) with a guard column 5C_18_-AR-II (4.6 mm i.d. × 10 mm, Nacalai Tesque) with methanol at the flow rate of 1.0 mL/min.

### Isolation of oxidized pigment 1

Oxidized *molischianum*-LH2 was concentrated by ultracentrifugation using Amicon centricon concentrators (50 kDa cutoff, Merck Millipore), followed by evaporation with a smart evaporator (BioChromato) in the dark. The pigment **1** was extracted from the resulting sample with methanol and purified by reverse-phase HPLC using a column 5C_18_-AR-II (10 mm i.d. × 250 mm, Nacalai Tesque) with methanol at the flow rate of 1.0 mL/min. VIS (methanol) λ_max_ 692 (relative intensity, 1.00), 443 (0.98), 411 nm (0.93); HRMS (APCI) found: *m/z* 909.5378, calcd for C_55_H_73_MgN_4_O_6_: MH^+^, 909.5375.

### Synthesis of 17,18-didehydro-BPhe *a*

17,18-Didehydro-BPhe *a* was synthesized from BPhe *a* by oxidation with FeCl_3_ according to previous reports^11,12^. A nitromethane solution of FeCl_3_⋅6H_2_O (4.0 eq.) was added to a dichloromethane solution of BPhe *a*, and the mixed solution was stirred at room temperature in the dark for 5 min. The reaction was quenched by addition of methanol and washed with distilled water. The organic layer was dried over anhydrous Na_2_SO_4_, followed by evaporation under reduced pressure. The residue was purified by silica gel flash column chromatography (Merck Kieselgel 60, 0–10% Et_2_O/CH_2_Cl_2_) to give the titled compound: mp 250 °C; VIS (CH_2_Cl_2_) λ_max_ 695 (relative intensity, 0.61), 649 (0.10), 553 (0.11), 521 (0.12), 419 nm (1.00); ^1^H NMR (600 MHz, CDCl_3_) δ 9.47 (1H, s, 20-H), 9.36 (1H, s, 5-H), 8.78 (1H, s, 10-H), 6.68 (1H, s, 13^2^-H), 5.28 (1H, br-t, *J* = 7 Hz, P2-H), 4.63 (2H, d, *J* = 7 Hz, P1-H_2_), 4.54 (1H, dq, *J* = 7, 3 Hz, 7-H), 4.29 (1H, dt, *J* = 8, 3 Hz, 8-H), 3.91–3.84 (2H, m, 17-CH_2_), 3.82 (3H, s, 13^2^-COOCH_3_), 3.68 (3H, s, 12-CH_3_), 3.60 (3H, s, 2-CH_3_), 3.27 (3H, s, 18-CH_3_), 3.22 (3H, s, 3^1^-CH_3_), 2.97, 2.84 (each 1H, ddd, *J* = 17, 10, 7 Hz, 17^1^-CH_2_), 2.49–2.42, 2.19–2.12 (each 1H, m, 8-CH_2_), 1.94–1.86 (2H, m, P3-CH_2_), 1.89 (3H, d, *J* = 7 Hz, 7-CH_3_), 1.63 (3H, s, P3-CH_3_), 1.55–1.44 (1H, P14-CH), 1.38–0.96 (18H, m, P4-(CH_2_)_2_CH(CH_2_)_3_CH(CH_2_)_3_), 1.13 (3H, t, *J* = 7 Hz, 8^1^-CH_3_), 0.87, 0.84 (each 3H, d, *J* = 7 Hz, P15-(CH_3_)_2_), 0.82, 0.81 (each 3H, d, *J* = 7 Hz, P7-, P11-CH_3_) [the two inner NH signals were invisible.]; HRMS (APCI) found: *m/z* 887.5680, calcd for C_55_H_75_N_4_O_6_: MH^+^, 887.5681.

### HPLC analysis of oxidized pigment 1 after demetallation

The central magnesium was removed from **1**, which was purified described above, by addition of an aliquot of hydrochloric acid in methanol. The demetallated pigment was quickly extracted with diethyl ether and dried over anhydrous Na_2_SO_4_, followed by dryness under stream of nitrogen gas. The residue was dissolved with methanol and eluted on a reverse-phase column 5C_18_-AR-II (6 mm i.d. × 250 mm, Nacalai Tesque) with methanol at the flow rate of 1.0 mL/min. The elution profile of this pigment was compared with that of the synthesized 17,18-didehydro-BPhe *a* under the same HPLC conditions.

### HPLC analysis of lycopene

LH2 proteins were concentrated by ultracentrifugation with Amicon centricon concentrators (50 kDa cutoff, Merck Millipore), followed by evaporation with a smart evaporator (BioChromato) in the dark. Lycopene was extracted from the samples with a mixture of methanol and dichloromethane (1/1, vol/vol), followed by evaporation under reduced pressure. The residues were dissolved with an HPLC eluent (hexane/ acetone = 99/1, vol/vol) and eluted on a normal-phase column 5SL-II (6 mm i.d. × 250 mm, Nacalai Tesque) with hexane/acetone (99/1, vol/vol) at the flow rate of 0.5 mL/min.

### AFM measurements

A 100 μL aliquot of oxidized *molischianum*-LH2 solution in 20 mM Tris buffer containing 0.02% DDM and 150 mM NaCl (pH 8.0) was placed onto a cleaved mica (SPI Supplies). After standing for 30 min at room temperature in the dark, the mica surface was rigorously rinsed with 20 mM Tris buffer containing 150 mM NaCl (pH 8.0). The oxidized *molischianum*-LH2 was then observed in 20 mM Tris buffer containing 150 mM NaCl (pH 8.0) by a laboratory-built frequency modulation AFM controlled by a commercially available AFM controller (ARC2, Asylum Research)^29^ with a silicon cantilever with gold coating of deflection side (160 AC-NG, MikroMasch), which had a nominal spring constant of 26 N/m. The typical resonance frequency and Q factor in an aqueous buffer solution are 120 kHz and 7, respectively.

### Size exclusion chromatography

SEC analysis was performed basically according to previous reports^21,30,31^. *Molischianum*-LH2 proteins were eluted on a HiPrep 16/60 Sephacryl S-300 HR column (GE Healthcare) with 20 mM Tris buffer containing 0.02% DDM and 150 mM NaCl (pH 8.0) at the flow rate of 0.4 mL/min.

### Transient absorption spectroscopy

Femtosecond time-resolved TA spectroscopy was done according to a previous report^19^ with a pair of noncollinear optical parametric amplifiers (NOPA) (TOPAS-white, Light-Conversion), pumped by a regeneratively amplified Ti:sapphire laser (Solstice, Spectra-Physics), as light sources. Output of one of the NOPAs was set at 700 nm for excitation of the Q_y_ band of the oxidized pigment in oxidized *molischianum*-LH2. A prism pair was used to pre-compress the pulses and the pulse duration at the sample position was 16 fs (fwhm), which was measured by the self-diffraction frequency-resolved optical gating (SD-FROG) method. The excitation intensity at the sample position was 20 μW (20 nJ), and the diameter of the focused laser beam was ca. 0.15 mm. The polarization between the pump and probe pulses was set at the magic angle by rotating the polarization of the pump pulse by a Berek compensator (Model 5540, New Focus). White-light supercontinuum (410–930 nm) was generated by focusing the output of another NOPA centered at 1100 nm into a rotating CaF_2_ window (thickness: 2 mm) and it was divided into probe and reference pulses. The probe pulse was focused into a rotating sample cell excited by the pump pulse, and the transmitted light was guided into a multichrometer (MSP1000-V, Unisoku). The reference pulse was directly guided into another multichrometer of the same type and the differential absorbance (ΔAbs) of the sample was calculated. The heterodyne-detected optical Kerr effect (HD-OKE) signal between the pump and the probe pulses was obtained by replacing the sample solution in the rotating cell with neat carbon tetrachloride, and the electronic response signal was used to compensate the group velocity dispersion of the TA signal. Oxidized *molischianum*-LH2 was solubilized in 20 mM Tris buffer containing 0.02% DDM (pH 8.0), and the Q_y_ absorbance of B850 BChl *a* of oxidized *molischianum*-LH2 was set at ca. 0.8–0.9 with the 2-mm optical length.

## Supplementary information

Supplementary Information 1.

## Data Availability

Data generated or analyzed during the current study are included in this published article and are available from the corresponding author on reasonable request.
