# Supplementary material for: In situ formation of photoactive B-ring reduced chlorophyll isomer in photosynthetic protein LH2
Source: Sci Rep. 2020 Nov 9;10:19383. doi: 10.1038/s41598-020-76540-1 (PMC7652862; doi:10.1038/s41598-020-76540-1)
Supplement: Supplementary file 1 — Supplementary Information 1. [file 41598_2020_76540_MOESM1_ESM.pdf]

Supporting Information

In situ formation of photoactive B-ring reduced chlorophyll isomer in photosynthetic protein LH2

Yoshitaka Saga [1,*], Yuji Otsuka [1], Daichi Funakoshi [2], Yuto Masaoka [2], Yu Kihara [2], Tsubasa Hidaka [2], Hiroka Hatano [3], Hitoshi Asakawa [3,4], Yutaka Nagasawa [2], & Hitoshi Tamiaki [2]

[1]Department of Chemistry, Faculty of Science and Engineering, Kindai University, Higashi-Osaka, Osaka 577-8502, Japan.

[2]Graduate School of Life Sciences, Ritsumeikan University, Kusatsu, Shiga 525-8577, Japan.

[3]Graduate School of Natural Science and Technology, Kanazawa University, Kanazawa 920-1192, Japan.

[4]Bio-AFM Frontier Research Center, Kanazawa University, Kanazawa 920-1192, Japan.

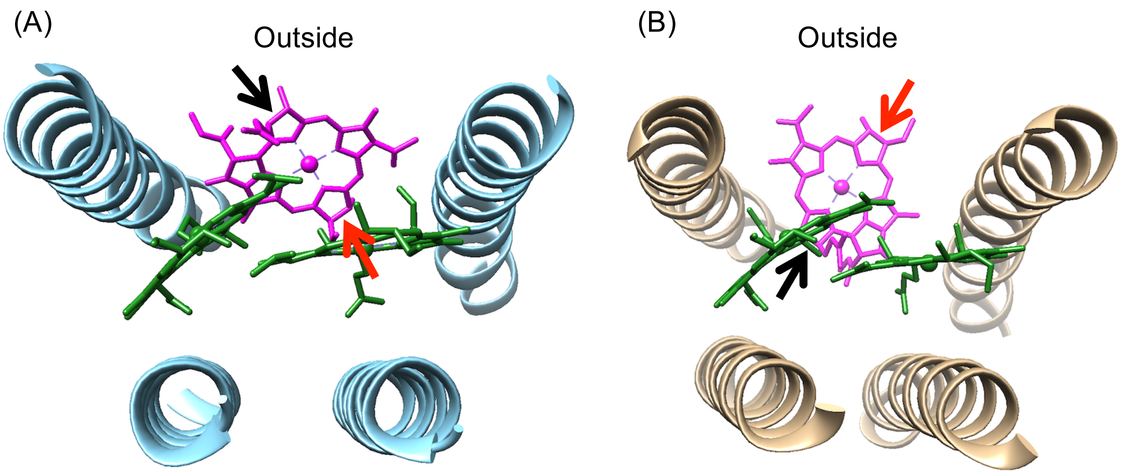

Figure S1. Top-view structures of the binding sites of B800 BChl *a* in *molischianum*-LH2 (A) and *acidophilus*-LH2 (B). B800 and B850 BChl *a* pigments are colored magenta and green, respectively. The phytyl esters of BChl *a* are omitted. The B- and D-rings in B800 BChl *a* are indicated by red and black arrows, respectively. Protein Data Bank Entries: 1LGH and 1NKZ for *molischianum*-LH2 and *acidophilus*-LH2, respectively. The protein structures were depicted using UCSF Chimera (http://www.cgl.ucsf.edu/chimera/) version 1.11.2.

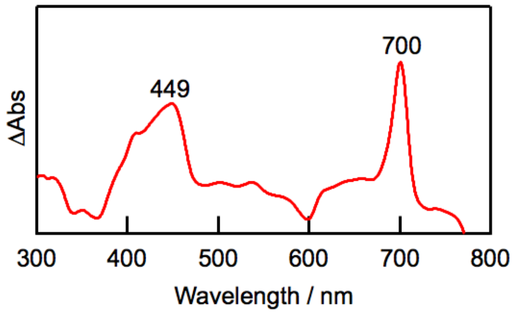

Figure S2. Difference spectrum obtained by subtracting the spectrum of native *molischianum*-LH2 (Fig. 2A) from oxidized *molischianum*-LH2 (Fig. 2B).

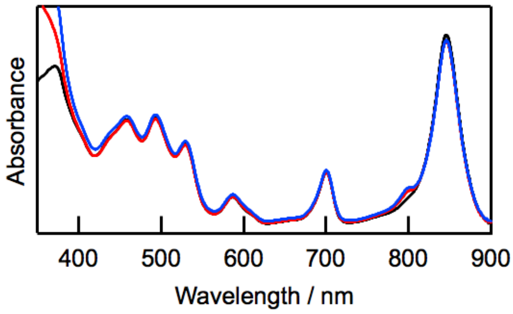

Figure S3. Spectral changes of oxidized *molischianum*-LH2 by incubation with sodium ascorbate (100 mM) in 20 mM Tris buffer containing 0.02% *n*-dodecyl-*β*-D-maltoside (pH 8.0) at room temperature for 0 h (black), 3 h (red), and overnight (blue).

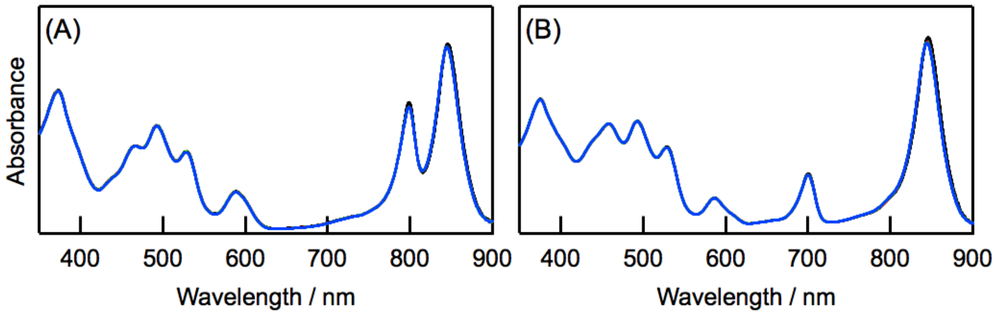

Figure S4. Spectral changes of native (A) and oxidized *molischianum*-LH2 (B) in 20 mM Tris buffer containing 0.02% *n*-dodecyl-*β*-D-maltoside (pH 8.0) at 40°C for 0 h (black), 1 h (red), 2h (green), and 3 h (blue). Note that all the spectra almost overlapped.

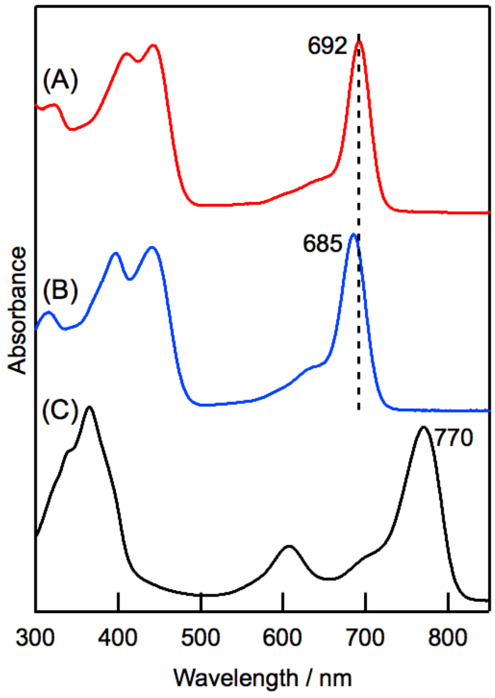

Figure S5. Electronic absorption spectra of oxidized pigment **1** (A), AcChl *a* (B), and BChl *a* (C) in methanol.

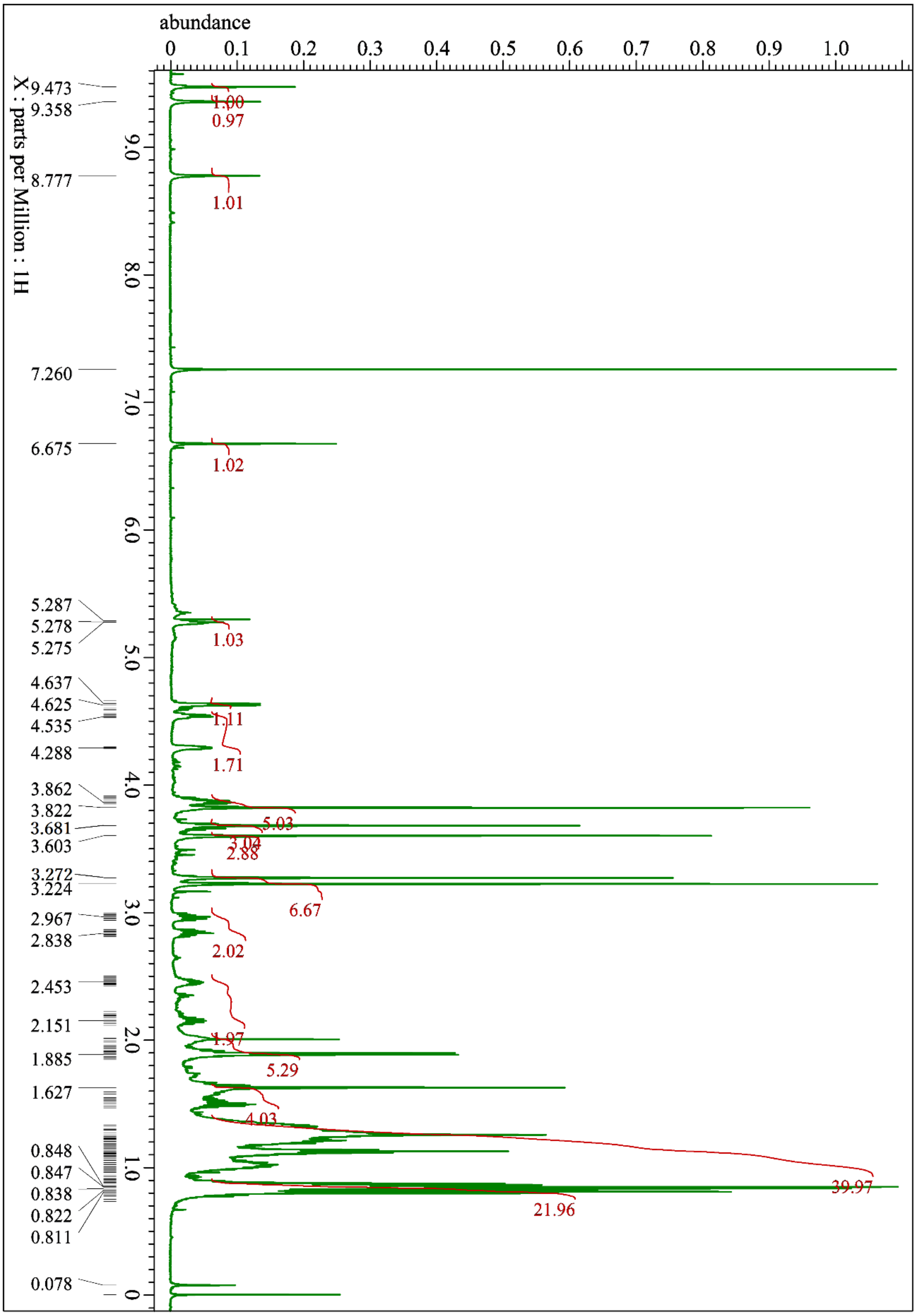

Figure S6. ^{1}H NMR spectrum of 17,18-didehydro-BPhe *a* synthesized from BPhe *a* in $CDCl_3$.

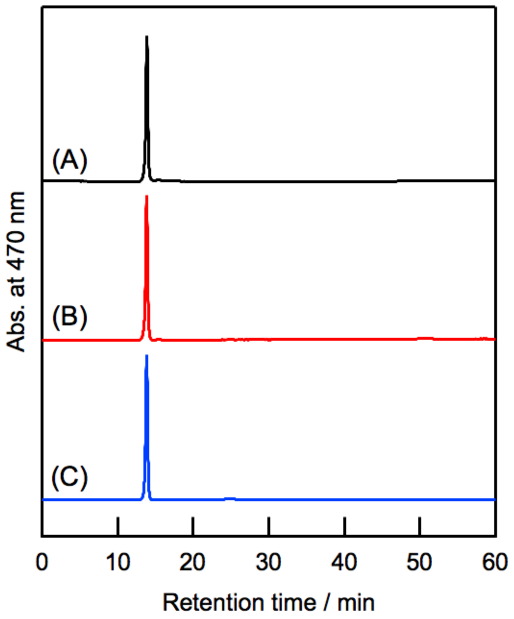

Figure S7. HPLC chromatograms of lycopene extracted from native (A) and oxidized *molischianum*-LH2 (B), and commercially available lycopene as a standard sample (C). The samples were eluted on a normal-phase column 5SL-II (6 mm i.d. × 250 mm, Nacalai Tesque) with hexane/acetone (99/1, vol/vol) at the flow rate of 0.5 mL/min.

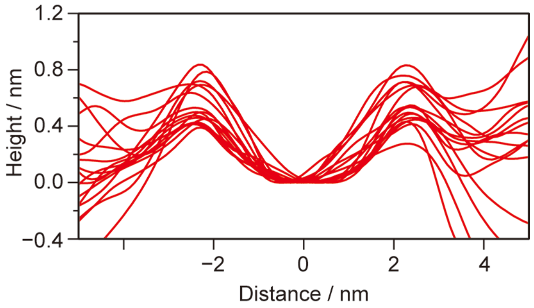

Figure S8. Overlapped height-profiles of 16 samples of oxidized *molischianum*-LH2 by FM-AFM.

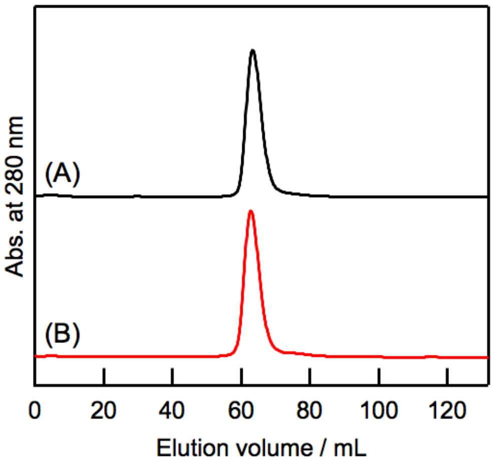

Figure S9. SEC chromatograms of native (A) and oxidized *molischianum*-LH2 (C). The samples were eluted on a HiPrep 16/60 Sephacryl S-300 HR column (GE Healthcare) with 20 mM Tris buffer containing 0.02% *n*-dodecyl-*β*-D-maltoside and 150 mM NaCl (pH 8.0) at the flow rate of 0.4 mL/min.

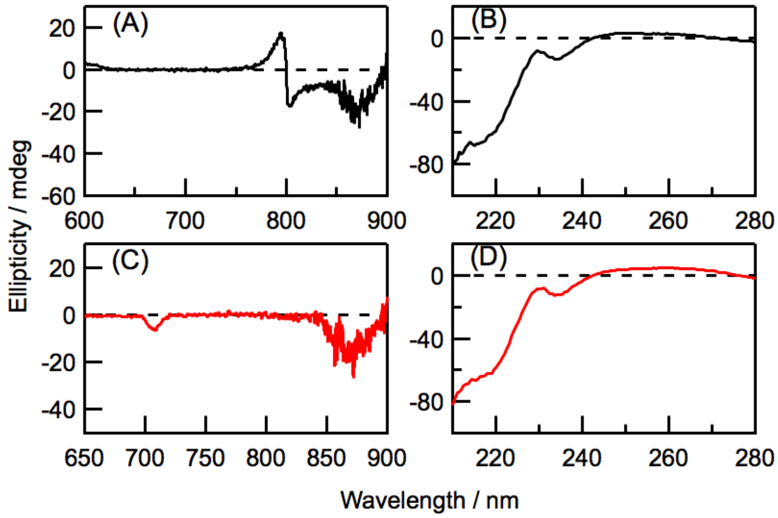

Figure S10. CD spectra of native (A/B) and oxidized *molischianum*-LH2 (C/D) in the Q_y region (A/C) and the UV-region (B/D) in 20 mM Tris buffer containing 0.02% *n*-dodecyl-*β*-D-maltoside (pH 8.0). The Q_y absorbance values of B850 BChl *a* in the LH2 samples used for the measurements were 0.8.

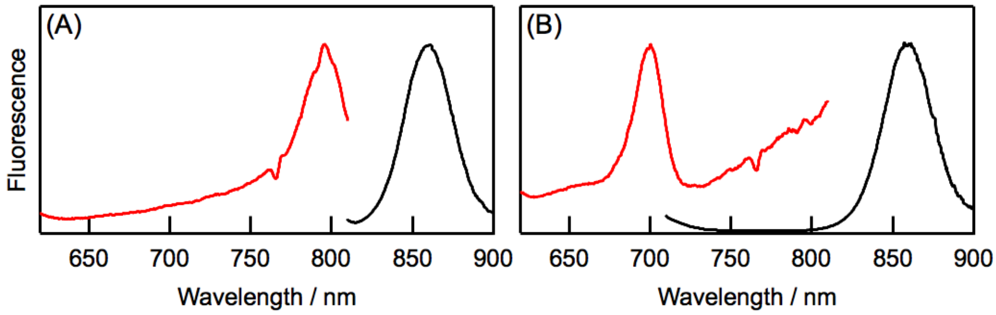

Figure S11. Fluorescence emission (black curves) and excitation spectra (red curves) of native (A) and oxidized *molischianum*-LH2 (B) in 20 mM Tris buffer containing 0.02% *n*-dodecyl-*β*-D-maltoside (pH 8.0). Excitation wavelengths in the measurements of emission spectra of native and oxidized *molischianum*-LH2 were 800 and 700 nm, respectively. Excitation spectra were obtained by detection at 860 nm.

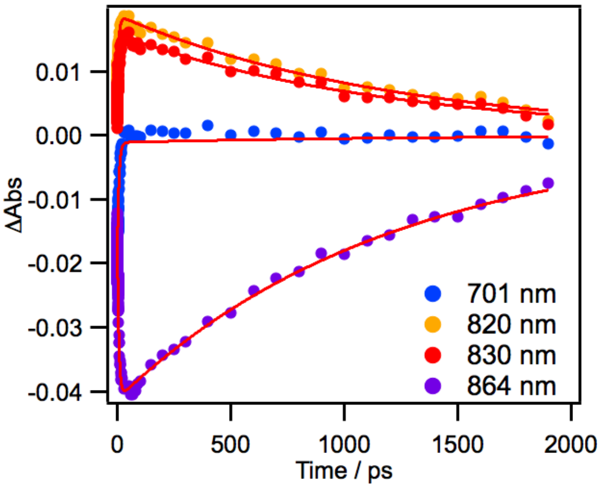

Figure S12. Time dependence of ΔAbs at various wavelengths (filled circles) and the results of global analysis (lines) for oxidized *molischianum*-LH2.
